# Association of Polymorphisms in PD-1 and LAG-3 Genes with Acute Myeloid Leukemia

**DOI:** 10.3390/medicina60050721

**Published:** 2024-04-26

**Authors:** Lamjed Mansour, Mashael Alqahtani, Ali Aljuaimlani, Jameel Al-Tamimi, Nouf Al-Harbi, Suliman Alomar

**Affiliations:** Department of Zoology, College of Science, King Saud University, Building 05, Riyadh 11451, Saudi Arabia; mashaelf93@hotmail.com (M.A.); alialhasani198@gmail.com (A.A.); jhattamimi@gmail.com (J.A.-T.); noufnalharbi@gmail.com (N.A.-H.); syalomar@ksu.edu.sa (S.A.)

**Keywords:** immune checkpoint molecules, PDCD-1, LAG-3, SNP polymorphisms, acute myeloid leukemia, middle east

## Abstract

*Background and objectives:* Acute myeloid leukemia (AML) is a hematological malignancy characterized by uncontrolled proliferation of immature myeloid cells. Immune checkpoint molecules such as programmed cell death protein 1 (PD-1) and lymphocyte activation gene-3 (LAG-3) are essential for controlling anti-tumor immune responses. This study aims to explore the correlation between specific genetic variations (SNPs) in the *PDCD1* (rs2227981) and *LAG3* (rs12313899) genes and the likelihood of developing AML in the Saudi population. *Material and methods*: total of 98 Saudi AML patients and 131 healthy controls were genotyped for the *PDCD1* rs2227981 and *LAG3* rs12313899 polymorphisms using TaqMan genotyping assays. A logistic regression analysis was conducted to evaluate the relationship between the SNPs and AML risk using several genetic models. *Results*: The results revealed a significant association between the *PDCD1* rs2227981 polymorphism and increased AML risk. In AML patients, the frequency of the G allele was considerably greater than in healthy controls (OR = 1.93, 95% CI: 1.31–2.81, *p* = 0.00080). The GG and AG genotypes were associated with a very high risk of developing AML (*p* < 0.0001). In contrast, no significant association was observed between the *LAG3* rs12313899 polymorphism and AML risk in the studied population. In silico analysis of gene expression profiles from public databases suggested the potential impact of PDCD1 expression levels on the overall survival of AML patients. *Conclusions*: This study provides evidence for the association of the PDCD1 rs2227981 polymorphism with an increased risk for AML in the Saudi population.

## 1. Introduction

Leukemia is a type of hematological cancer defined by the abnormal growth and accumulation of immature white blood cells, called blasts, which displace normal blood cell production. Leukemias are categorized into myeloid or lymphoid subtypes based on cellular lineage and disease development, and can manifest as acute or chronic [1]. They start in the bone marrow from hematopoietic stem cells and progenitor cells. In 2020, leukemia was the fifteenth most commonly diagnosed cancer globally, with 474,519 cases and 311,594 deaths, making it the eleventh leading cause of death from malignant diseases [2]. Leukemia rates in Saudi Arabia have risen in recent years. The number of cases in 2018 reached roughly 437,033. In 2020, there were 27,885 new cases reported [3]. The cause of acute leukemia, including acute lymphoblastic leukemia (ALL) and acute myeloid leukemia (AML), is yet unidentified. Genome-wide association studies pinpointed two chromosomal regions, 7p12.2 and 10q21.2, containing risky single nucleotide polymorphisms (SNPs) in study participants of Caucasian, Asian, and African descent [4,5,6,7]. AML primarily affects adults and is frequently found in the older population [8,9]. Shallis, et al. [10] states that patients exhibit chromosomal abnormalities at the time of diagnosis. Despite advancements in diagnosis and treatment, poor outcomes are still common in people with AML [11]. Conventional treatments for leukemia include chemotherapy, radiation therapy, and stem cell transplantation. Emerging therapies such as targeted therapies and immunotherapies are demonstrating potential [12,13]. Monoclonal antibodies that target leukemic cell surface antigens or regulate immune checkpoints like PD-1 are producing favorable outcomes against different types of leukemia [14]. These innovative treatments function by accurately eliminating cancer cells or stimulating the body’s innate anti-tumor defenses. AML is linked to several levels of risk, ranging from favorable to unfavorable, according to comprehensive national cancer networks [15]. Genetic alterations play a crucial role in acute myeloid leukemia (AML) [4]. The latest classifications from the World Health Organization (WHO) identify AML with biallelic mutations of CEBPA as a separate group with a positive outlook [16]. The immune system plays a vital role in surveilling tumors and inhibiting the advancement of leukemia. Several vital immunological molecules either boost or suppress anti-leukemia immune responses [17]. Antigen-presenting cells display tumor antigens to stimulate tumor-specific T cells via the T cell receptor and co-stimulatory molecules such as CD28 [18]. Activated T cells release cytokines to target and eliminate leukemia cells. Leukemic cells can evade immunological elimination by upregulating the production of inhibitory ligands that bind to immune checkpoint receptors on T cells. Increased expression of programmed death ligand 1 (PD-L1) inhibits T cell proliferation by binding to the programmed cell death protein 1 (PD-1) receptor [19,20]. Leukemia causes an increase in MHC class II molecules, which triggers the LAG-3 receptor on T cells, resulting in the suppression of lymphocyte activation [21]. PD-1 and LAG-3 are immune checkpoint receptors that are essential for regulating peripheral T cell tolerance and influencing anticancer immunological responses [22]. Single-nucleotide polymorphisms (SNPs) in PD-1 and LAG-3 genes are linked to vulnerability and medical results in different types of cancer [23,24,25]. In hematological malignancies, PD-1 and LAG-3 genetic variations have been associated with a risk and the prognosis of leukemia [23,26]. Their actions are believed to occur via modifying the structure, expression levels, or signaling activity of immune checkpoint proteins, therefore influencing the equilibrium between tumor immune evasion and immunological-mediated disease control [26]. Delving deeper into these genetic connections could offer valuable insights into the processes of immune evasion in hematological malignancies. This study aimed to investigate the correlation between SNPs of two inhibitory immune checkpoint receptors *PDCD1* rs2227981 and *LAG3* rs12313899 and the susceptibility to acute myeloid leukemia in the Saudi population.

## 2. Materials and Methods

### 2.1. Patients and Healthy Individuals

The study involved 98 Saudi patients diagnosed with de novo acute myeloid leukemia (AML), comprising 44 females (44.90%) and 54 males (55.10%) recruited from King Khaled Hospital in Riyadh. AML patients were diagnosed following WHO 2017 criteria with a thorough investigation involving a complete blood count, bone marrow examination, and flow cytometry. Chromosomal and fluorescent in situ hybridization (FISH) testing was conducted to confirm the existence of AML, in addition to other procedures. None of these patients started treatment before sampling. Blood samples were collected from AML patients between April 2018 and February 2020. To ensure the analysis only included cases of AML, patients with diagnoses of other malignancies or chronic conditions were excluded from the study. Almost 30% of patients were recruited from the pediatric department.

The control group comprised 131 healthy volunteers, with 49 females (37.40%) and 82 males (62.60%), who were matched for age and sex. The average age of the study participants was 27.56 ± 18.59 for those with AML and 29.35 ± 18.91 for the healthy control group. No control subjects had a personal or family history of AML or any other chronic or immunological disorders.

The study methods involving human volunteers were conducted in conformity with the Ethics Committee of the Faculty of Medicine at King Khaled Hospital (Ref. No. 20/0800/IRB). The procedures were conducted in accordance with the 1964 Helsinki statement, and all participants gave written informed consent.

### 2.2. DNA Extraction

Three milliliters of blood were collected under sterile conditions from each participant and preserved at −20 °C in tubes containing ethylenediaminetetraacetic acid (EDTA) for analysis. Peripheral blood DNA from AML patients and healthy controls was isolated using the QIAamp DNA Mini Kit (Qiagen, Hilden, Germany) following the manufacturer’s guidelines. The DNA concentration was determined using a Nanodrop ND-2000c spectrophotometer (Thermo Scientific in Wilmington, DE, USA).

### 2.3. SNP Selection and Genotyping Method

Two single-nucleotide polymorphisms (SNPs) in the immune checkpoint genes PDCD1 (rs2227981) and LAG3 (rs12313899),were selected using the dbSNP databases available at https://www.ncbi.nlm.nih.gov/snp/, accessed on 13 February 2023. SNPs were chosen based on having a minor allele frequency (MAF) of at least 5%. The Hardy–Weinberg equilibrium (HWE) *p*-value threshold was set at greater than 0.005, as shown in Table 1. Genotyping was conducted using the allelic discrimination method using VIC and FAM labels. TaqMan assays were ordered from Applied Biosystems and used as per the manufacturer’s instructions on an ABI Prism 7500 real-time PCR system. Real-time PCR was conducted in a 10 μL reaction system comprising 0.26 μL 2× SNP Genotyping Assay, 5.5 μL 2× Power Taq MasterMix Mix, 2.24 μL Nuclease-Free Water, and 2 μL DNA template (100 ng/μL). The PCR protocol for rs2227981 and rs12313899 involved an initial denaturation step at 95 °C for 10 min, followed by 40 cycles of denaturation at 95 °C for 15 s, annealing at 55 °C for 30 s, extension at 72 °C for 30 s, and a final extension at 72 °C for 5 min. For genotype confirmation and quality control, the genotyping was repeated for 5% of randomly chosen samples.

### 2.4. In Silico Analysis of Gene Expression Profiles and Their Correlation with Survival Prognosis

Gene expression profiles and patient survival data were analyzed using the Gene Expression Profiling Interactive Analysis (GEPIA) website, which is based on the TCGA transcription database created by Peking University. A total of 173 cases were compared to 70 controls from TCGA and GTEx datasets [27]. An analysis was conducted to examine the impact of gene expression levels (high vs. low) of PDCD1 and LAG3 on the overall survival rate of acute myeloid leukemia (AML) patients. The analysis was carried out using the Kaplan–Meier plot tool in the UALCAN database (http://ualcan.path.uab.edu/, accessed on 30 January 2024). Co-expression analysis of PDCD1 and LAG3 was predicted using the cBio portal cancer genomic online platform (https://www.cbioportal.org/, accessed on 16 February 2024) with Pearson and Spearman correlation coefficients.

### 2.5. Statistical Analysis

Alleles and genotypes’ relative risk was assessed through odds ratios (ORs) with a 95% confidence interval (CI) using five inheritance models: co-dominant, dominant, recessive, over-dominant, and log-additive. The analysis was conducted with the web-based SNPStats software program by Solé, et al. [28] accessed on 25 January 2024. SNPs were assessed for divergence from the Hardy–Weinberg equilibrium using the Chi-square test. The significance criterion for association was set at *p* < 0.05. The *p* value was corrected for multiple comparisons using Bonferroni correction to Pc 0.003. A total of 98 eligible patients and 131 controls were genotyped for four specific target SNPs: PD1–5 A>G (rs2227981) and LAG3 A>G (rs12313899). All genotypes adhered to a Hardy–Weinberg equilibrium.

## 3. Results

### 3.1. Association of PDCD1 (rs2227981A>G) Polymorphisms with AML

Comparative distributions of the *PD1-5 A>G* genotypes for the five examined inheritance models and alleles are reported in Table 2. Our results indicated that the *PD1-5* polymorphism in exon5 was associated with a very high risk for AML. The frequency of the G allele was significantly higher in patients compared to healthy group (OR: 1.93; 95% CI: 1.31–2.81 and *p* = 0.0008). Very strong associations were found in codominant, dominant, overdominant and additive models (*p* < 0.0001), which show an overall association of the G allele and GG and AG genotypes with AML. In the codominant model, the risk of AML is 15.76 times higher (95% CI: 5.43–45.78) for people with the AG genotype and 7.29 times higher (95% CI: 1.83–29.00) for people with the GG genotype. The AA genotype could be considered as highly protective against AML. Based on the AIC parameter, the codominant model could be the most appropriate one.

### 3.2. Association of LAG3 (rs12313899A>G) Polymorphisms with AML

The comparative distribution of the rs12313899A>G polymorphism in the intron position of *LAG3* gene between patient and control groups for all examined models is reported in Table 3. Our results indicate that this examined polymorphism was not associated with risk for AML (*p* > 0.05).

### 3.3. Stratification Analysis by Gender and Age

The potential association between the *PDCD1* rs2227981 and *LAG3* rs12313899 polymorphisms and risk of acute myeloid leukemia (AML) was studied through stratified analyses based on gender and age. A stratified analysis by gender showed no correlations between genotype at these polymorphic sites and risk of AML among males or females (Table 4). Similarly, a stratified analysis by age failed to identify any associations between genotype and AML risk (Table 5). Both polymorphisms did not show differential risk associations according to gender or age subgroups for AML.

### 3.4. In Silico Analysis of mRNA Differential Expression and Prognosis

**Figure 1:**
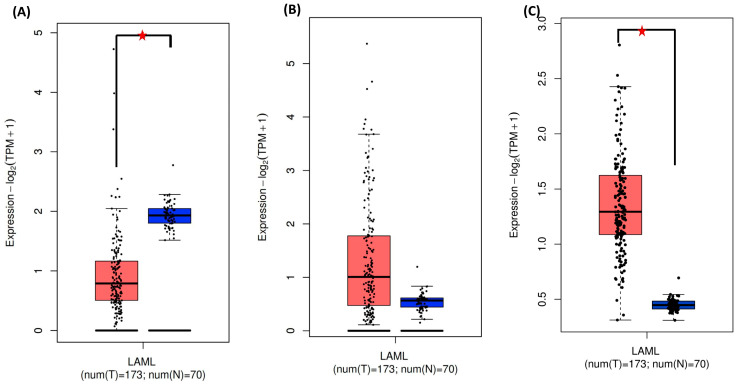
Expression of PDCD-1 (**A**) and LAG-3 (**B**) in the blood of AML patients (red) and healthy controls (blue). (**C**) mRNA signature of exhausted T cells in AML patients (red) and healthy controls (blue). Each bar indicates the average level of expression. (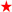) significant difference. Data were obtained from the GEPIA database (http://gepia.cancer-pku.cn, accessed on 14 February 2024).

An in silico analysis of mRNA differential expression and the prognostic impact in AML was performed using the GEPIA databases. The GEPIA database compared PDCD1 and LAG3 mRNA expression between AML tumor samples and healthy control samples from TCGA. PDCD1 mRNA levels were markedly reduced in AML samples in comparison to controls (Figure 1A). A great dispersion in the level of expression was observed among AML samples.

For the LAG3 mRNA expression, no significant difference between AML and control groups was observed, noting a dispersion of values among AML patients as reported for the PDCD1 gene (Figure 1B). On the other hand, analysis of the mRNA signature of exhausted T cells shows a higher level of the expression of PDCD1 and LAG3 among AML patients compared to controls (Figure 1C).

**Figure 2:**
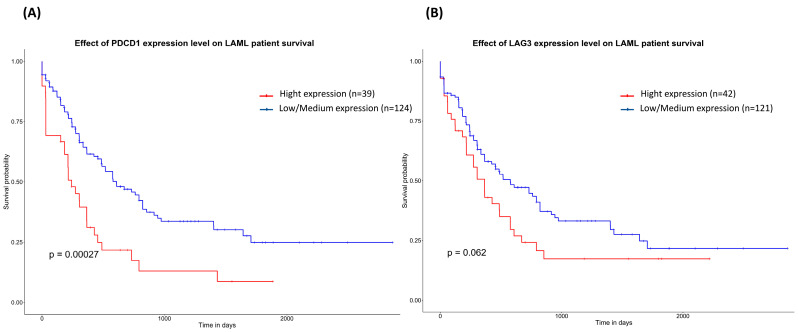
Kaplan–Meier graphs show the overall survival prognosis of AML patients based on hight or low PDCD1 mRNA expression (**A**) and LAG3 mRNA expression (B). Patients with expression above the median are shown in red, and patients with expression below the median are shown in blue. Kaplan–Meier analysis plots were obtained from the UALCAN database (http://ualcan.path.uab.edu/, accessed on 14 FEbruary 2024).

A Kaplan–Meier survival analysis in GEPIA evaluated the prognostic impact of PDCD1 and LAG3 mRNA expression. A higher PDCD1 expression is correlated with a significantly worse overall survival in AML patients (Figure 1B, *p* = 0.00027), indicating its potential use as a prognostic biomarker. However, LAG3 mRNA levels showed no significant association with overall survival (*p* = 0.06) (Figure 2B).

**Figure 3:**
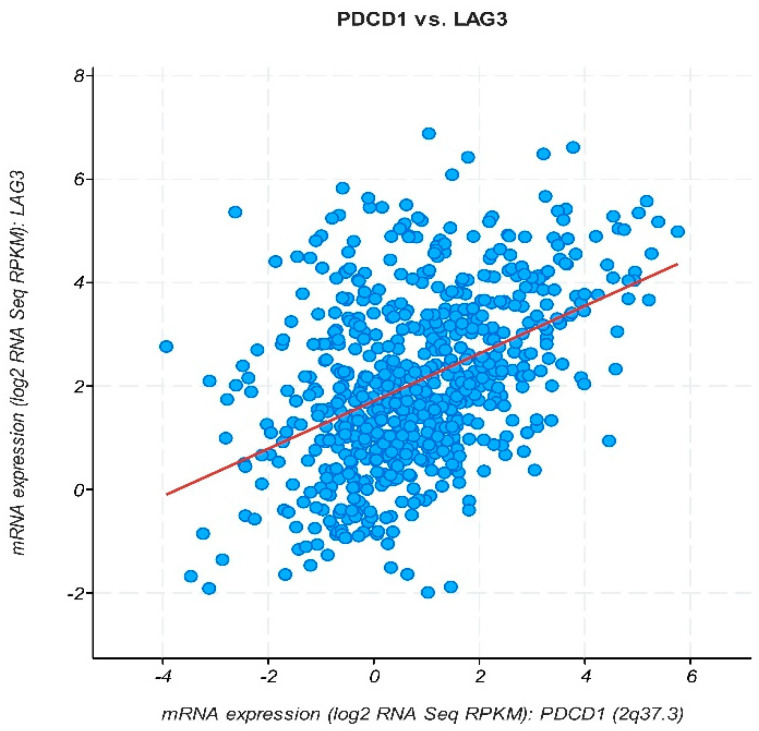
Co-expression of PDCD-1 and LAG-3 genes as determined by cBioPortal.

The cBioPortal database was used to analyze correlations between PDCD1 and LAG3 expressions among AML samples. A strong positive correlation was observed, with Pearson and Spearman correlation coefficients both being 0.43 (*p* < 0.0001) (Figure 3). This suggests a coordinated regulation of PDCD1 and LAG3 during AML development and progression.

## 4. Discussion

Acute myeloid leukemia (AML) is a heterogeneous hematological malignancy characterized by uncontrolled proliferation and impaired differentiation of myeloid progenitor cells. Despite advancements in diagnostic and therapeutic approaches, the prognosis for AML patients remains poor, with a high rate of relapse and resistance to conventional treatments. The immune system plays a vital role in tumor surveillance and elimination, and dysregulation of immune checkpoint pathways can contribute to tumor immune evasion and disease progression. In this study, we investigated the relationship between specific single-nucleotide polymorphisms (SNPs) in the *PDCD1* (rs2227981) and *LAG3* (rs12313899) genes encoding the immune checkpoint receptors PD-1 and LAG-3, respectively and the risk of developing AML in the Saudi population. Our findings revealed a significant correlation between the PDCD1 rs2227981 polymorphism and a higher risk for AML; however, no significant correlation was found for the LAG3 rs12313899 polymorphism.

The PDCD1 rs2227981 G allele, situated in exon 5 of the PD-1 gene, was found to be more prevalent in AML patients than in healthy controls, indicating its possible involvement in regulating immune evasion mechanisms in leukemia. The codominant and log-additive models exhibited the most robust relationships, suggesting a dose-dependent impact of the G allele on AML risk. The AA genotype seemed to provide protection against AML formation, reinforcing the potential functional significance of this polymorphism. The expression of the PD-1 receptor is highly regulated. It is mainly expressed on activated T cells, playing a critical role in regulating peripheral tolerance and preventing autoimmunity. Upon binding to its ligands, PD-L1 and PD-L2, which are expressed on tumor cells and antigen-presenting cells, PD-1 signaling inhibits T cell proliferation, cytokine production, and cytotoxic activity. Leukemic cells often upregulate PD-L1 expression, leading to T cell exhaustion and suppression of anti-tumor immune responses [19,20].

The rs2227981 polymorphism is situated on exon 5 at position +7785 A/G. Multiple studies have reported significant associations with various types of solid cancers, including lung cancer [29], cervical cancer [30], and breast cancer [31]. A recent meta-analysis suggested that the PD-1.5 (rs2227981) polymorphism is linked to significantly reduced cancer risks in individuals with the A allele compared to those with the G allele [31,32,33,34]. In addition, the role of polymorphisms in PD-1 genes was investigated in several types of hematological cancer studies.

Several studies have assessed the potential link between PDCD1 gene variations and hematological cancers such as multiple myeloma and leukemia. Kasamatsu, et al. [35] did not find any link between individual examined SNPs (PD-1.1, rs41386349, PD-1.9) and multiple myeloma. However, an association was observed with specific haplotypes (GCC/GCC) of the three selected SNPs with disease. Similarly, Grzywnowicz, et al. [36] observed no links between five specific SNPs of PDCD1, including the PD-1.5 polymorphism, and the risk of developing chronic lymphocytic leukemia in a population from Poland. Recently, Wu et al. [23] reported an association between rs2227982 and AML in Jinan in China, but no link was found for rs2227981. It is worth noting that rs2227981 is a synonymous polymorphism that does not alter the protein’s final amino acid structure. The strong association between this polymorphism and malignancies is likely due to a linkage disequilibrium with other PD-1 gene polymorphisms, which could potentially change the expression level of PD-1. In contrast to the findings for PD-1 rs2227981, our study did not observe a significant association between the LAG3 rs12313899 polymorphism and AML risk in the Saudi population. The LAG-3 receptor, expressed on activated T cells and natural killer (NK) cells, negatively regulates T cell activation and proliferation upon binding to its ligands, including MHC class II molecules [37,38]. While our study did not find a significant association between the LAG3 rs12313899 polymorphism and AML risk, a few other studies have reported associations between LAG3 genetic variations and susceptibility to or prognosis of some cancer types, including AML [23], where LAG3 rs2365094 was associated with risk stratification for AML in a Chinese population from Ji’nan, Shandong. A protective association with progression of localized prostate cancer was reported for LAG3:rs1997510 in non-Hispanic white men [39]. In addition, Lee et al. [40] showed an association between LAG-3 rs2365094G> C and the risk of multiple myeloma in women, while they reported a protective effect of the A allele of LAG-3 rs3782735.

In addition to the genetic association analysis, we performed in silico analyses to evaluate the potential impact of PDCD1 and LAG3 gene expression levels on the overall survival of AML patients and their association with AML. Based on a GEPA database analysis, the expression of PD-1 is higher in the control than in AML. This result is to be taken with caution for many reasons. First, the two analyzed groups have different sizes (173 vs. 70) and matching between these two groups for age, sex and ethnicity is not guaranteed. In addition, we noticed that the expression level values in the AML plot are more dispersed, suggesting a heterogeneity in the clinical profile of patients that could be related to the stage of disease, medication, age, gender, genome profile, and other factors. All these factors could influence the differential expression of PD-1 genes among individuals with cancer diseases [41]. On the other hand, our analysis shows a potential correlation between high PD-1 mRNA levels and a decreased overall survival. This result confirms the heterogeneity of the AML group, although the specific mechanisms underlying this observation require further investigation.

PD-1 expression has been observed on B and T cells and other immune system cells, in addition to elevated expressions of PD-1 and its ligand PD-L1 in hematological malignancies [42]. When PD-1 interacts with its ligand in cancerous cells, it reduces the sensitivity of these cells to immune responses, allowing them to grow and develop [43]. Thus, it has been reported that increased PD-1 signaling is associated with a worse overall survival in AML patients [44]. Studies indicate that PD-1 signaling could impact the progression and unfavorable outcome of AML by promoting T cell exhaustion. Upregulation of LAG-3 has been reported in various malignancies, including AML, and is associated with T cell dysfunction and tumor immune evasion [45,46]. Radwan et al. [45] showed that the upregulation LAG-3 expressions was correlated with patients with an unfavorable prognosis of AML compared with those with a favorable prognosis. It is important to note that the functional consequences of the examined SNPs in the PDCD1 and LAG3 genes have not been fully elucidated. SNPs can potentially influence gene expression, protein structure, or protein–protein interactions, ultimately affecting immune checkpoint regulation and anti-tumor immune responses.

## 5. Conclusions

Further functional studies are warranted to elucidate the underlying mechanisms by which these polymorphisms may contribute to AML susceptibility or prognosis. In addition to the several strengths of our study, there are also limitations to consider. First, the sample size was relatively small, which may have limited the statistical power to detect modest associations, particularly for the LAG3 polymorphism. Larger multicenter studies with diverse ethnic populations would be beneficial to validate and extend our findings. Second, we focused on two specific SNPs in the PDCD1 and LAG3 genes, but additional genetic variations or haplotype analyses may provide further insights into the potential role of these immune checkpoint genes in AML susceptibility and prognosis. Finally, it is essential to consider the complex interplay between genetic factors, environmental exposures, and lifestyle factors in the development and progression of AML. Future studies should integrate comprehensive genetic profiling, epigenetic analyses, and detailed clinical and epidemiological data to gain a more holistic understanding of the interplay between immune checkpoint pathways and leukemogenesis.

## Figures and Tables

**Table 1:** Characteristics of specific genetic variations related to the PDCD1 rs2227981 and LAG3 rs12313899 SNPs.

Gene	SNP ID/Assay ID	Chromosome Position	Nucleotide Change	Region	MAF in Human Populations				
					Global	European	East Asian	American	Qatari
*PDCD1*	rs2227981	Chr2/241851121	A>G	Exon5	0.35	0.40	0.27	0.44	0.33
*LAG3*	rs12313899	Chr 12/6768692	A>G	Intro	0.47	0.39	0.43	0.42	0.40

MAF: minor allele frequency.

**Table 2:** Association between PD1–5 rs2227981 allele and genotype frequencies and AML.

Locus	Model	Genotype	Control (%) *n* = 132	AML *n* = 98	OR (95% CI)	*p*-Value	AIC
*PDCD1*	Allele	A	0.65	0.48	1	0.0008	
		G	0.35	0.52	1.93 (1.31–2.81)		
	Codominant	AA	50 (38.2%)	4 (4.1%)	1	<0.0001	274.2
		AG	69 (52.7%))	87 (88.8%)	15.76 (5.43–45.78)		
		GG	12 (9.2%)	7 (7.1%)	7.29 (1.83–29.00)		
	Dominant	AA	50 (38.2%)	4 (4.1%)	1	<0.0001	284
		AG + GG	81 (61.8%)	94 (95.9%)	14.51 (5.02–41.91)		
	Recessive	AA + AG	119 (90.8%)	91 (92.9%)	1	0.58	316.4
		GG	12 (9.2%)	7 (7.1%)	0.76 (0.29–2.01)		
	Overdominant	G/G + A/A	62 (47.3%)	11 (11.2%)	1	<0.0001	280.1
		A/G	69 (52.7%)	87 (88.8%)	7.11 (3.48–14.52)		
	Log-Additive				3.35 (1.90–5.91)	<0.0001	296.1

AML: acute myeloid leukemia, OR: odds ratio, CI: 95% confidence interval, AIC: Akaike information criterion, all *p* < 0.0031 were considered significant.

**Table 3:** Association between *LAG3* rs12313899 alleles and genotype frequencies and AML.

Locus	Model	Genotype	Control (%) *n* = 132	AML *n* = 98	OR (95% CI)	*p*-Value	AIC
*LAG3*	Allele	A	0.54	0.63	1	0.23	
		G	0.46	0.37	1.163 (0.907–1.492)		
	Codominant	AA	44 (34.1%)	41 (41.8%)	1	0.2	323.5
		AG	52 (40.3%)	41 (41.8%)	0.85 (0.47–1.53)		
		GG	33 (25.6%)	16 (16.3%)	0.52 (0.25–1.08)		
	Dominant	AA	44 (34.1%)	41 (41.8%)	1	0.23	319.9
		AG + GG	85 (65.9%)	57 (58.2%)	0.72 (0.42–1.24)		
	Recessive	AA + AG	96 (74.4%)	82 (83.7%)	1	0.09	318.4
		GG	33 (25.6%)	16 (16.3%)	0.57 (0.29–1.10)		
	Overdominant	AA + GG	77 (59.7%)	57 (58.2%)	1	0.82	321.2
		AG	52 (40.3%)	41 (41.8%)	1.07 (0.62–1.82)		
	Log-Additive		---	---	0.74 (0.52–1.05)	0.091	311.6

AML: acute myeloid leukemia, OR: odds ratio, CI: 95% confidence interval, AIC: Akaike information criterion, all *p* < 0.0031 were considered significant.

**Table 4:** Correlation between *PDCD1* rs2227981 and *LAG3* rs12313899 polymorphisms and AML susceptibility after gender stratification.

Locus	Model	Genotype	AML Female *n* = 44	AML Male *n* = 54	OR (95% CI)	*p*-Value	AIC
*PDCD1* *rs2227981A>G*	Allele	A	0.5	0.57		0.77	
		G	0.5	0.47	0.89 (0.50–1.57)		
	Codominant	AA	2 (4.5%)	5 (9.3%)	1		
		AG	40 (90.9%)	47 (87%)	0.47 (0.09–2.56)	0.352	139.9
		GG	2 (4.5%)	2 (3.7%)	0.40 (0.03–5.15)		
	Dominant	AA	2 (4.5%)	5 (9.3%)	1	0.36	138.1
		AG + GG	42 (95.5%)	49 (90.7%)	0.47 (0.09–2.53)		
		AA + AG	42 (95.5%)	52 (96.3%)	1	0.83	138.8
	Recessive	GG	2 (4.5%)	2 (3.7%)	0.81 (0.11–5.98)		
	Overdominant	AA + GG	4 (9.1%)	7 (13%)	1	0.54	138.5
		AG	40 (90.9%)	47 (87%)	0.67 (0.18–2.46)		
	Log-Additive		40 (90.9%)	47 (87%)	0.67 (0.18–2.46)	0.51	138.4
*LAG3* rs12313899A>G		A	0.63	0.62		1	
	Alleles	G	0.37	0.38	1.02 (0.56–182)		
		AA	17 (38.6%)	24 (44.4%)	1		
	Codominant	AG	21 (47.7%)	20 (37%)	0.67 (0.28–1.61)	0.55	139.6
		GG	6 (13.6%)	10 (18.5%)	1.18 (0.36–3.87)		
		AA	17 (38.6%)	24 (44.4%)	1	0.56	138.5
	Dominant	AG + GG	27 (61.4%)	30 (55.6%)	0.79 (0.35–1.77)		
		AA + AG	38 (86.4%)	44 (81.5%)	1	0.51	138.4
	Recessive	GG	6 (13.6%)	10 (18.5%)	1.44 (0.48–4.33)		
	Overdominant	AA + GG	23 (52.3%)	34 (63%)	1	0.29	137.7
		AG	21 (47.7%)	20 (37%)	0.64 (0.29–1.45)		
	Log-Additive				0.98 (0.56–1.71)	0.95	138.8

AML: acute myeloid leukemia, OR: odds ratio, 95% CI: 95% confidence interval, AIC: Akaike information criterion, all *p* < 0.0031 were considered significant.

**Table 5:** Correlation between *PDCD1* rs2227981 and *LAG3* rs12313899 polymorphisms and AML susceptibility after age stratification.

Locus	Model	Genotype	AML Age < 29 *n* = 52	AML Age ≥ 29 *n* = 42	OR (95% CI)	*p*-Value	AIC
*PDCD1* *rs2227981A>G*	Allele	A	54 (52%)	47 (51%)	1	1	
		G	50 (48%)	45 (49%)	0.96 (0.55–1.69)		
	Codominant	AA	2 (4.5%)	5 (9.3%)	1		
		AG	40 (90.9%)	47 (87%)	0.47 (0.09–2.56)	0.352	139.9
		GG	2 (4.5%)	2 (3.7%)	0.40 (0.03–5.15)		
	Dominant	AA	2 (4.5%)	5 (9.3%)	1	0.36	138.1
		AG + GG	42 (95.5%)	49 (90.7%)	0.47 (0.09–2.53)		
	Recessive	AA + AG	42 (95.5%)	52 (96.3%)	1	0.83	138.8
		GG	2 (4.5%)	2 (3.7%)	0.81 (0.11–5.98)		
	Overdominant	AA + GG	4 (9.1%)	7 (13%)	1	0.54	138.5
		AG	40 (90.9%)	47 (87%)	0.67 (0.18–2.46)		
	Log-Additive		40 (90.9%)	47 (87%)	0.67 (0.18–2.46)	0.51	138.4
*LAG3* rs12313899A>G		A	0.66 (69)	0.59 (54)	1	0.30	
	Alleles	G	0.34 (35)	0.41 (38)	0.72 (0.40–1.28)		
	Codominant	AA	4 (7.7%)	3 (6.5%)	1	0.97	141.4
		AG	46 (88.5%)	41 (89.1%)	1.19 (0.25–5.63)		
		GG	2 (3.8%)	2 (4.3%)	1.33 (0.11–15.70)		
	Dominant	AA	4 (7.7%)	3 (6.5%)	1	0.82	139.4
		AG + GG	48 (92.3%)	43 (93.5%)	1.19 (0.25–5.64)		
	Recessive	AA + AG	50 (96.2%)	44 (95.7%)	1	0.9	139.5
		GG	2 (3.8%)	2 (4.3%)	1.14 (0.15–8.41)		
	Overdominant	AA + GG	6 (11.5%)	5 (10.9%)	1	0.92	139.5
		AG	46 (88.5%)	41 (89.1%)	1.07 (0.30–3.77)		
	Log-Additive		---	---	1.16 (0.35–3.83)	0.8	139.4

AML: acute myeloid leukemia, OR: odds ratio, CI: 95% confidence interval, AIC: Akaike information criterion, all *p* < 0.0031 were considered significant.

## Data Availability

All data relevant to the study are included in the article.
